# Determining Factors for Independent Walking in Patients Undergoing Cardiovascular Surgery: Differences between Coronary Artery Bypass Grafting, Heart Valve Surgery, and Aortic Surgery

**DOI:** 10.3390/healthcare9111475

**Published:** 2021-10-30

**Authors:** Yui Sakamoto, Yosuke Morimoto, Masatoshi Hanada, Yudai Yano, Terumitsu Sawai, Takashi Miura, Kiyoyuki Eishi, Ryo Kozu

**Affiliations:** 1 Department of Cardiopulmonary Rehabilitation Science, Nagasaki University Graduate School of Biomedical Sciences, Nagasaki 852-8520, Japan; bb55317019@ms.nagasaki-u.ac.jp (Y.S.); morimoto@reha.kobegakuin.ac.jp (Y.M.); mstshnd@nagasaki-u.ac.jp (M.H.); yudai-121@nagasaki-u.ac.jp (Y.Y.); sawai@nagasaki-u.ac.jp (T.S.); 2 Department of Physical Therapy, Faculty of Rehabilitation, Kobe Gakuin University, Kobe 651-2180, Japan; 3 Cardiorespiratory Division, Department of Rehabilitation Medicine, Nagasaki University Hospital, Nagasaki 852-8501, Japan; 4 Department of Cardiovascular Surgery, Nagasaki University Hospital, Nagasaki 852-8501, Japan; takashirsa@nagasaki-u.ac.jp (T.M.); keishi@nagasaki-u.ac.jp (K.E.)

**Keywords:** cardiac rehabilitation, thoracic surgery, cardiac surgical procedures, aortic disease, walking, mobility limitation, early ambulation, postoperative care

## Abstract

Physical deconditioning often occurs during the acute phase after cardiovascular surgery, and unassisted walking is required to achieve independence, to manage cardiac diseases, and to prevent recurrences. This study aims to investigate the characteristics of independent walking after cardiovascular surgery. We conducted a retrospective cohort study in patients who underwent cardiovascular surgeries (total of 567 patients): 153 in the coronary artery bypass grafting (CABG) group, 312 in the heart valve surgery group, and 102 in the aortic surgery group. We evaluated the effect of each surgery group on the cardiac rehabilitation (CR) progression. The factors associated with independent walking were age, renal diseases, intensive care unit (ICU) length of stay, and post-operative respiratory complications in the CABG group. In the heart valve surgery group, the factors were New York Heart Association functional classification, renal and respiratory diseases, ICU length of stay, duration of mechanical ventilatory support, and post-operative cardiovascular and respiratory complications. In the aortic surgery group, these were ICU length of stay and acute kidney injury. The CR progression in patients who underwent aortic surgery was significantly longer than those who underwent CABG and heart valve surgery (*p* < 0.001). New intervention strategies are needed for patients with prolonged ICU stays.

## 1. Introduction

Early achievement of independent walking after cardiovascular surgery plays an important role in independence in daily living, management of cardiac disease, and prevention of the recurrence of disease [1]. It is recommended that cardiac rehabilitation (CR) after cardiovascular surgery should be started in the early post-operative period, within 24–72 h after surgery [1,2,3]. Early CR is effective in reducing post-operative complications, in improving exercise tolerance, and in preventing delirium [1,4,5,6,7,8]. In addition, independent walking is targeted to be achieved within 4–7 days after surgery, and continued exercise training, mainly aerobic exercise such as walking, is effective in reducing rehospitalization and coronary risk factors and in enhancing quality of life [1,9,10,11,12,13,14,15]. However, patients who have undergone cardiovascular surgery are often subjected to prolonged immobilization [16,17]. Surgery and subsequent prolonged bed rest can lead to rapid deterioration of the musculoskeletal system [18,19]. Monteleone et al. [20] reported that more than half of patients after cardiothoracic surgery had physical dysfunction and that 25.5% of patients were unable to walk independently at discharge. Independent walking after cardiovascular surgery has received attention as an important acquired behavior in previous studies [16,17,20].

Saitoh et al. [16] showed that post-operative renal function and fluid balance were independent factors that determine walking ability in the early post-operative period in patients undergoing coronary artery bypass grafting (CABG) and valvular heart disease. Kato et al. [17] identified age, serum albumin levels, operative time, and post-operative atrial fibrillation as independent predictors of walking delay during CR in patients who underwent heart valve surgery. After aortic surgery, bed rest was likely to be a priority, and there were significantly fewer prior studies on CR than in other cardiac fields [12] and no prior studies on the determinants of independent walking. Additionally, post-aortic surgery is a major indication for CR [1,8,12,13], as the number of such surgeries is increasing every year [21] and post-operative survival rates are also rising [22]. Aortic surgery, which is highly invasive, is expected to cause significant impairment in walking independence in the early post-operative period due to complications and other deconditioning factors [12,23,24,25].

The current limitation in the literature is that studies examining the factors that determine independent walking are limited to the type of surgery. Increased knowledge of the determinants of independent walking may be useful for appropriate post-operative CR and resource management, and early independent walking may contribute to shorter hospital stays and lower medical costs [4,5,6,7,8]. Therefore, the aims of this study were (1) to investigate the characteristics of delayed achievement of independent walking after different types of cardiovascular surgery and (2) to evaluate the CR progression in different surgical techniques.

## 2. Materials and Methods

### 2.1. Study Design

This retrospective cohort study was conducted at a single center. We enrolled patients who underwent CR after cardiovascular surgery at Nagasaki University Hospital between June 2013 and December 2016. This study was approved by the Nagasaki University Hospital Clinical Research Ethical Committee (reference number 15061113). Informed consent was obtained in the form of an opt-out option on the website; those who were not interested were excluded.

### 2.2. Subjects

We included patients who underwent CR after all cardiovascular surgeries, except surgery for abdominal aortic aneurysms and lower extremity bypass surgery. Of them, the exclusion criteria for patients in this study were being under 18 years of age at the time of surgery, having underwent combined cardiac and aortic surgery, repeated surgery or surgery with general anesthesia within one-month, post-operative stroke, and death in the hospital after surgery. Patients who were unable to walk before or after cardiovascular surgery, even with the use of a walking aid (e.g., cane, crutches, walker), were also excluded.

### 2.3. Data Collection

Using an electronic medical record system, we obtained data on the demographics of the patients from hospital admission to discharge. The pre-operative data were age, sex, body mass index (BMI), New York Heart Association (NYHA) functional classification, cardiac function (left ventricular ejection fraction: LVEF, the ratio of mitral peak velocity of early filling to early diastolic mitral annular velocity: E/e’), pre-operative diagnosis, and comorbidities. Comorbidities, including hypertension [26], diabetes [27], renal disease (chronic kidney disease), respiratory disease (chronic obstructive pulmonary disease, interstitial pneumonia, and bronchial asthma, and pneumoconiosis), and central nervous system disease (stroke and brain tumor), were diagnosed by the cardiovascular surgeon. 

The intra-operative data included operative time, amount of bleeding, aortic cross clumping time, cardiopulmonary bypass time, and the required number of emergency surgeries. The post-operative data included intensive care unit (ICU) length of stay, duration of mechanical ventilation, complications, progression of CR, and the overall length of stay in the hospital. The duration of mechanical ventilation was defined as the time from immediately after the surgery to successful extubation (no re-intubation within 24 hours). Post-operative complications, either as a newly developed or an exacerbated comorbidity disease, until the completion of independent walking for up to one month, were diagnosed by the cardiovascular surgeon or intensivist. These were defined as follows: cardiovascular complications (ischemic heart disease, infective endocarditis, pericardial effusion, bradyarrhythmia, supraventricular arrhythmia, and ventricular arrhythmia) treated with more than local anesthesia such as cardiac catheterization, replacement of existing drains, placement or puncture of new drains into the pericardial cavity, pacemaker implantation, acute kidney injury (AKI) [28], delirium [29], and respiratory complications (upper and lower airway infections, pneumonia, respiratory failure, atelectasis, and pneumothorax) [30,31]. For respiratory infections, we followed the criteria described by Canet [30]. Respiratory failure was defined as being on mechanical ventilation for >48 hours after the surgery [31].

### 2.4. Early Post-Operative CR Program

The CR program was based on the Japanese Circulation Society’s early post-operative CR guidelines as follows: (1) initiated on the post-operative day 1, number of sessions at 1–2 times/day, the frequency at 6 times/week daily, and continued until discharge; (2) carried out under the supervision of a physical therapist; and (3) consisted of passive and active limb exercises, respiratory physical therapy, and early mobilization (sitting on the edge of a bed, standing, walking, and exercise training) [1].

Progression during early post-operative CR was analyzed using the following criteria: the initiation of mobilization, including sitting on the edge of the bed and standing at the bedside; initial walking; and the completion of 200 m walking. In accordance with the Japanese Circulation Society’s early post-operative CR guidelines, we defined 200 m walking as achieving independent walking and as the goal of early post-operative CR [1].

### 2.5. Statistical Analyses

The distribution of the data was analyzed using the Shapiro–Wilk test. When the data were not normally distributed, a non-parametric test was used. Demographics and peri-operative data of patients, for each type of surgery, were compared by one-way analysis of variance; if the results were significant, multiple comparisons with Bonferroni correction were performed. Simple and multiple linear regression analyses were performed to assess the factors related to independent walking for each type of surgery. The variables identified as significant (*p* < 0.05) on single linear regression analysis were used as independent variables for multiple regression analyses, considering multicollinearity. Pearson product-moment correlation was used to examine the relationship between the ICU length of stay and progression of CR. Values are expressed as median (interquartile range) or number (%). The level of statistical significance was set at *p <* 0.05. All statistical analyses were performed using JMP® 15.0 (SAS Institute Inc., Cary, NC, USA).

## 3. Results

The study cohort initially consisted of 763 consecutive patients who underwent cardiovascular surgery during the study period. Of them, patients who met the exclusion criteria were as follows. Patients who underwent combined cardiac and aortic surgery (*n* = 80), who could not walk after surgery until discharge (*n* = 32), who died during hospitalization (*n* = 23), who could not walk before surgery (*n* = 17), who had post-operative stroke (*n* = 17), who were under 18 years of age (*n* = 10), who underwent repeat cardiovascular surgery within one month (*n* = 10), and who underwent other surgeries within one month (*n* = 7). Consequently, a total of 567 patients were included in the study.

### 3.1. Demographics of Patients

The peri-operative demographics of patients are shown in Table 1. The demographics were classified as follows: CABG (*n* = 153 (on-pump, *n* = 80; off-pump, *n* = 73)), heart valve surgery (*n* = 312 (minimally invasive cardiac surgery, *n* = 120)), and aortic surgery (*n* = 102). The pre-operative diagnosis for CABG was angina pectoris (*n* = 133), asymptomatic myocardial ischemia (*n* = 12), and myocardial infarction (*n* = 8). The pre-operative diagnosis for heart valve surgery was valvular insufficiency (*n* = 165), valvular stenosis (*n* = 84), combination of valvular insufficiency and stenosis (*n* = 23), combined valvular disease (*n* = 18), infectious endocarditis (*n* = 7), and others, including chronic heart failure and intra-cardiac thrombosis (*n* = 15). The pre-operative diagnosis for aortic surgery was aortic aneurysm (*n* = 34), dissociative aortic aneurysm (*n* = 32), acute aortic dissection (*n* = 19), aortic aneurysm rupture (*n* = 5), and others, including infection of artificial blood vessels and aortic root disease (*n* = 12).

### 3.2. Post-Operative Progresssion of CR

The post-operative progression during CR, according to the type of surgery, is shown in Table 1. The initiation of sitting on the edge of a bed and standing at the bedside was not significantly different between CABG and heart valve surgery patients but was significantly achieved later in those with aortic surgery (*p* < 0.001). Initial walking and independent walking were significantly delayed in heart valve surgery, CABG, and aortic surgery patients (*p* < 0.05), in that given order.

### 3.3. Factors Related to Post-Operative Independent Walking

The results of the determining factors related to independent walking, for each surgery, on simple linear regression analysis and multiple linear regression analysis are shown in Table 2 and Table 3, respectively. As a result of multiple linear regression analysis, the determining factors in the CABG group were age, renal disease as a comorbidity, ICU length of stay, and post-operative respiratory complications. In the heart valve surgery group, the determining factors were NYHA functional classification, renal disease and respiratory disease as comorbidities, ICU length of stay, duration of mechanical ventilatory support, and post-operative cardiovascular and respiratory complications. In the aortic surgery group, the only determining factors were the ICU length of stay and post-operative AKI. ICU length of stay was a determining factor for all three types of surgery.

### 3.4. Relationships between ICU Length of Stay and Progression of CR in the Aortic Surgery Group

**Figure 1:**
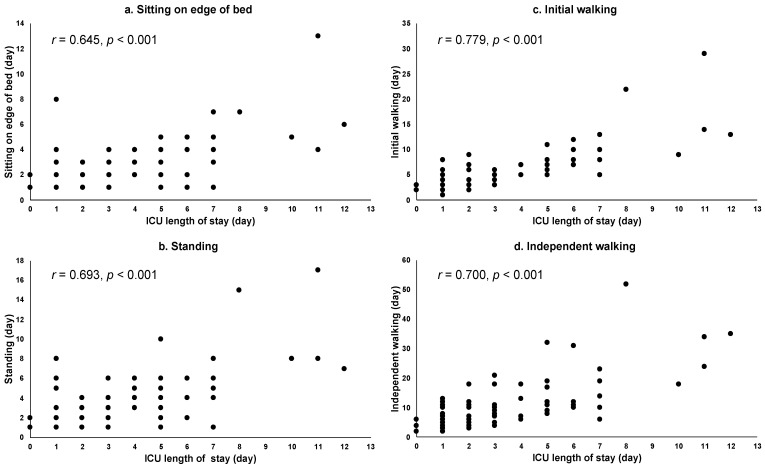
Relationships between ICU length of stay and CR in aortic surgery group. Abbreviation: intensive care unit: ICU. Notes: (**a**) correlation between ICU length of stay and achieving sitting on the edge of a bed, (**b**) correlation between ICU length of stay and achieving standing, (**c**) correlation between ICU length of stay and achieving initial walking, and (**d**) correlation between ICU length of stay and achieving independent walking.

The relationship between the ICU length of stay and the progression of CR in patients who underwent aortic surgery is shown in Figure 1. The ICU length of stay was positively correlated with progression during CR (initial walking (*r* = 0.779, *p* < 0.001), independent walking (*r* = 0.700, *p* < 0.001), standing (*r* = 0.693, *p* < 0.001), and sitting on the edge of a bed (*r* = 0.645, *p* < 0.001)).

## 4. Discussion

The main findings of the present study were as follows: (1) the ICU length of stay was a common determining factor for the influence of independent walking in all three groups; (2) post-operative respiratory complications were a common determining factor for independent walking in the CABG and valve groups; (3) post-operative AKI was a determining factor for independent walking only in the aortic surgery group; and (4) progression of CR in patients with aortic surgery was significantly later than it was in the CABG and heart valve surgery patients. These results indicate that there are both common and specific factors associated with post-operative independent walking in each of the three surgical groups. To our knowledge, this is the first study to examine in detail the determining factors for independent walking according to different types of cardiovascular surgery.

According to the results of our study, the ICU length of stay was an important factor in determining independent walking after the cardiovascular surgery, regardless of the surgical type. A previous study reported that 47% of ICU patients could not be mobilized because of respiratory and hemodynamic instability, problems with staffing, and a busy ICU environment [32]. In this study, the ICU length of stay was correlated with both independent walking and the progress of early post-operative CR. These findings suggest that patients in the ICU for a long period were not only in poor post-operative condition but also do not walk and spend less time in mobility because of the ICU-specific environment.

Factors determining independent walking were extracted as post-operative respiratory complications in the CABG group and the valve surgery group. Post-operative respiratory complications in the aortic surgery group were not a determining factor for independent walking but had the highest incidence (65%) among the three surgical groups. Canet et al. [30], which was referred to for the definition of post-operative respiratory complications in this study, reported a post-operative incidence of 39.6%, which was similar to the incidence in the CABG and valvular surgery groups of this study. Post-operative respiratory complications have been reported to influence increased mortality, prolonged hospital stay, and progression of CR [30,33]. The higher incidence of post-operative respiratory complications in the aortic surgery group compared with the other two surgery groups suggests that more patients were affected by their post-operative general condition than in the other two surgery groups, which may have contributed to the overall delay in CR for aortic surgery compared with the other two surgery groups.

AKI was extracted as an independent factor determining independent walking in the aortic surgery group. In previous studies, cardiopulmonary bypass time is independently associated with an increased risk of post-operative AKI in patients after the aortic surgery [23]. Similar to earlier studies, we also observed a longer cardiopulmonary bypass time in patients with aortic surgery, compared with the CABG and heart valve surgery patients. Post-operative AKI has also been reported to cause a delay in CR due to a longer critical care period after cardiac surgery [16]. Specifically, the management of AKI requires early dialysis or continuous hemodiafiltration for kidney damage and various complications. These may have a direct impact on the early progression of CR, including independent walking.

In previous studies [16,17], pre- and intra-operative factors were associated with achieving independent walking in patients who underwent CABG and heart valve surgery; these findings, with regard to CABG and heart valve surgery, were consistent with the results of our study. However, in the aortic surgery group, post-operative factors such as length of ICU stay and post-operative AKI, but not pre-operative factors, were associated with independent walking. In addition, in the aortic surgery group, pre-operative factors as determinants of independent walking were not extracted as a significant factor in the single regression analysis. These findings indicate that the post-operative factors may play an important role in walking during CR after aortic surgery. The post-operative factors in the aortic surgery group were the significantly longer operative time and greater amount of bleeding, an increase in the number of emergency surgeries, the longer duration of mechanical ventilatory support, and a higher incidence of complications compared with the other two surgery groups. This indicates that aortic surgery is more invasive than the other two surgical groups, which may be the cause of the longer post-operative functional recovery.

In this study, the progression of CR in the enrolled patients was in accordance with that observed in previous studies [1,16,17]. Compared with the other two surgical groups, CR progression in the aortic surgery group was significantly delayed from sitting on the edge of a bed to independent walking. The length of hospital stay was also the longest among the three surgical groups. These results suggest that aortic surgery, compared with other cardiac surgeries, is more likely to interfere with post-operative recovery. As an approach to early recovery of physical functions, it has been reported that interventions such as pre-operative rehabilitation and pre-operative education are effective in improving post-operative physical and mental functions in CABG and valve surgery [34,35,36]. However, because aortic surgery often involves emergency surgery associated with aneurysm rupture or dissection and because there is a risk of rupture even in cases that are asymptomatic and discovered incidentally [37], it is often impossible to take measures such as rehabilitation intervention pre-operatively. Therefore, it is difficult for CR in the aortic surgery group to achieve similar physical recovery with the same interventions as the other two surgeries, suggesting the need for more extensive post-operative interventions.

The present study has several limitations. First, our study was retrospective and conducted at a single center. Thus, the external validity was insufficient. There may be some bias in the progression of CR, and larger multicenter studies may provide additional insight into CR after cardiovascular surgery. Second, the pre-operative physical function was not assessed in this study; therefore, the association of progress of CR with prior physical function could not be studied. Future research will be needed to evaluate pre-and post-operative physical function.

A strength of this study was that it included patients with more extended demographics compared with previous studies; we identified multiple factors that contributed to delayed independent walking after cardiovascular surgery, including aortic surgery.

## 5. Conclusions

Independent walking is a fundamental activity after discharge from cardiovascular surgery to prevent a recurrence, to continue exercise, and to live an independent daily life. In this study, we showed that there are factors common to the three surgical groups and factors specific to each surgical group that determine post-operative independent walking. In particular, aortic surgery showed delayed post-operative recovery compared with the other two surgical groups, suggesting the need for a more generous support and approach than conventional CR. Further studies are needed to determine how to tailor interventions to the characteristics of each cardiovascular surgery.

## Figures and Tables

**Table 1:** Demographics and peri-operative data of patients.

Variables	CABG Group (*n* = 153)		Heart Valve Surgery Group (*n* = 312)		Aortic Surgery Group (*n* = 102)		*p* Value ^a^
Age, year	69.0 [63.0–77.5]		70.0 [60.0–77.0]		69.0 [58.0–78.0]		0.205
Male, n (%)	115 (75.2)		155 (49.7)	^†††^	67 (65.7)	^‡^	<0.001
BMI, kg/m^2^	23.8 [21.6–26.5]	*	22.4 [20.2–24.7]	^†††^	23.2 [20.7–24.8]		<0.001
NYHA functional classification, n (%)							
I/II/III/IV/Unknown	43 (28.1)/45 (29.4)/39 (25.5)/24 (15.7)/2 (1.3)		72 (23.1)/109 (34.9)/93 (29.8)/36 (11.5)/2 (0.6)		53 (52.0)/20 (19.6)/10 (9.8)/4 (3.9)/15 (14.7)	^‡‡‡^	<0.001
LVEF, %	65.5 [54.8–72.3]		66.0 [58.0–72.0]		66.0 [59.0–70.0]		0.700
E/e’, cm/s	10.6 [8.0–13.7]		14.3 [10.6–20.4]	^†††^	9.0 [7.6–12.5]	^‡‡‡^	<0.001
Comorbidities, n (%)							
Hypertension	90 (58.8)		133 (42.6)	^††^	64 (62.7)	^‡‡^	<0.001
Diabetes	62 (40.5)	***	55 (17.6)	^†††^	7 (6.9)	^‡^	<0.001
Renal disease	28 (18.3)		66 (21.2)		11 (10.8)		0.058
Respiratory disease	25 (16.3)		59 (18.9)		14 (13.7)		0.471
Central nervous system disease	28 (18.3)		32 (10.3)	^†^	10 (9.8)		0.040
Operative time, min	304.0 [248.5–340.5]	***	257.5 [221.5–294.8]	^†††^	381.0 [321.8–457.0]	^‡‡‡^	<0.001
Amount of bleeding, mL	1010.0 [500.0–1649.5]	***	1230.0 [545.5–1800.0]		2290.0 [980.0–3495.0]	^‡‡‡^	<0.001
Aortic cross clumping time, min	0.0 [0–74.5]	***	80.0 [68.0–108.0]	^†††^	135.0 [102.3–169.5]	^‡‡‡^	<0.001
Cardiopulmonary bypass time, min	72.0 [0–119]	***	135.0 [111.0–166.0]	^†††^	207.0 [161.8–246.5]	^‡‡‡^	<0.001
Emergency surgery, n (%)	22 (14.4)	***	5 (1.6)	^†††^	50 (49.0)	^‡‡‡^	<0.001
Length of intensive care unit stay, day	1.0 [1.0–2.0]	***	1.0 [0–2.0]		2.0 [1.0–5.0]	^‡‡‡^	<0.001
Duration of mechanical ventilatory support, hour	11.8 [8.6–17.9]	***	9.6 [7.4–14.9]	^†††^	18.8 [11.8–25.1]	^‡‡‡^	<0.001
Post-operative complications, n (%)	62 (40.52)	***	116 (37.2)		71 (69.6)	^‡‡‡^	<0.001
Cardiovascular	13 (8.6)		40 (12.8)		10 (9.8)		0.372
Acute kidney injury	2 (1.3)		9 (2.9)		7 (6.9)		0.052
Delirium	11 (7.2)		20 (6.4)		13 (12.7)		0.135
Respiratory	50 (32.7)	***	79 (25.3)		67 (65.7)	^‡‡‡^	<0.001
Cardiac rehabilitation progress							
Sitting on the edge of a bed, day	1.0 [1.0–2.0]	***	1.0 [1.0–1.0]		2.0 [1.0–3.0]	^‡‡‡^	<0.001
Standing, day	1.0 [1.0–2.0]	***	1.0 [1.0–2.0]		2.0 [1.0–4.0]	^‡‡‡^	<0.001
Initial walking, day	3.0 [2.0–4.0]	***	2.0 [2.0–4.0]	^††^	4.0 [3.0–6.3]	^‡‡‡^	<0.001
Independent walking, day	5.0 [4.0–7.0]	***	5.0 [3.0–6.0]	^†^	8.0 [5.0–12.0]	^‡‡‡^	<0.001
Length of stay in hospital, day	18.0 [15.0–21.0]	***	18.0 [15.0–24.0]		23.5 [19.0–30.0]	^‡‡‡^	<0.001

Abbreviation: BMI: Body Mass Index, CABG: Coronary Artery Bypass Graft, E/e’: The ratio of mitral peak velocity of early filling to early diastolic mitral annular velocity, LVEF: Left Ventricle Ejection Fraction, NYHA: New York Heart Association. Notes: Data are presented as median [quartile] and number (%). ^a^ *p* Value by one-way analysis of variance. Multiple comparisons test: CABG (vs. Aortic surgery): * *p* < 0.05, *** *p* < 0.001, Valve replacement and angioplasty (vs. CABG): † *p* < 0.05, †† *p* < 0.01, ††† *p* < 0.001, Aortic surgery (vs. Valve replacement and angioplasty): ‡ *p* < 0.05, ‡‡ *p* < 0.01, ‡‡‡ *p* < 0.001. CABG included on-pump CABG *n* = 80, off pump CABG *n* = 73. Heart valve surgery included cardiovascular surgery *n* = 192, minimally invasive cardiac surgery *n* = 120.

**Table 2:** Single liner regression analysis for determining factors for achieving independent walking.

	CABG Group (*n* = 153)		Heart Valve Surgery Group (*n* = 312)		Aortic Surgery Group (*n* = 102)	
	β [95%CI]	*p* Value	β [95%CI]	*p* Value	β [95%CI]	*p* Value
Age, year	0.109 [0.046–0.173]	<0.001	0.074 [0.019–0.128]	0.009	0.023 [−0.101–0.147]	0.714
Male (presence)	−0.138 [−0.941–0.665]	0.735	−0.453 [−1.202–0.296]	0.235	−0.021 [−1.751–1.709]	0.981
BMI, kg/m^2^	−0.217 [−0.414–0.020]	0.031	−0.209 [−0.431–0.012]	0.064	−0.314 [−0.170–0.797]	0.201
NYHA functional classification, class	0.501 [−0.158–1.160]	0.135	1.467 [0.692–2.243]	<0.001	−0.184 [−1.933–1.565]	0.835
LVEF, %	−0.021 [−0.079–0.038]	0.486	−0.075 [−0.131–0.019]	0.008	0.098 [−0.082–0.278]	0.098
E/e’, cm/s	0.205 [0.039–0.370]	0.016	0.063 [−0.019–0.144]	0.130	0.301 [−0.149–0.752]	0.187
Comorbidity (presence)						
Hypertension	−0.260 [−0.964–0.444]	0.466	−0.465 [−1.222–0.291]	0.227	0.791 [−0.889–2.471]	0.352
Diabetes	0.201 [−0.506–0.907]	0.576	0.044 [−0.941–1.028]	0.930	−1.215 [−4.425–1.995]	0.454
Renal disease	1.615 [0.756–2.475]	<0.001	2.279 [1.396–3.161]	<0.001	2.433 [−0.146–5.012]	0.064
Respiratory disease	1.014 [0.089–1.939]	0.032	1.365 [0.419–2.310]	0.001	1.035 [−1.397–3.468]	0.400
Central nervous system disease	0.719 [0.171–1.610]	0.113	0.086 [−1.151–1.322]	0.892	1.321 [−1.403–4.046]	0.334
Operative time, min	0.006 [−0.004–0.016]	0.215	0.012 [0.001–0.022]	0.025	0.031 [0.020–0.041]	<0.001
Amount of bleeding, mL	0.001 [8.377–0.002]	0.048	0.001 [−0.001–0.001]	0.584	0.001 [0.001–0.001]	<0.001
Aortic cross clumping time, min	0.008 [−0.009–0.024]	0.364	0.020 [−0.002–0.042]	0.074	0.036 [0.007–0.065]	0.017
Cardiopulmonary bypass time, min	0.001 [−0.003–0.019]	0.158	0.005 [−0.008–0.018]	0.436	0.024 [0.005–0.043]	0.014
Emergency surgery (presence)	2.069 [1.137–3.000]	<0.001	0.739 [−2.247–3.725]	0.627	1.490 [−0.118–3.098]	0.069
Length of intensive care unit stay, day	1.428 [1.136–1.713]	<0.001	2.137 [1.958–2.316]	<0.001	2.261 [1.801–2.721]	<0.001
Duration of mechanical ventilatory support, hour	0.047 [0.018–0.076]	0.002	0.152 [0.135–0.168]	<0.001	0.182 [0.071–0.293]	0.001
Post-operative complications (presence)						
Cardiovascular	1.318 [0.087–2.548]	0.036	2.698 [1.617–3.779]	<0.001	3.375 [0.722–6.028]	0.013
Acute kidney injury	1.265 [−1.784–4.314]	0.414	2.282 [0.055–4.508]	0.045	7.534 [4.688–10.381]	<0.001
Delirium	1.394 [0.069–2.719]	0.039	2.834 [1.335–4.332]	<0.001	3.154 [0.796–5.513]	0.009
Respiratory	2.107 [1.449–2.765]	<0.001	2.785 [1.981–3.590]	<0.001	2.671 [1.037–4.304]	0.002
Types of surgery (presence)						
Off-pump CABG	−0.507 [−1.198–0.183]	0.148				
MICS			−1.338 [−2.094–−0.581]	<0.001		

Abbreviation: BMI: Body Mass Index, CABG: Coronary Artery Bypass Graft, E/e’: The ratio of mitral peak velocity of early filling to early diastolic mitral annular velocity, LVEF: Left Ventricle Ejection Fraction, MICS: minimally invasive cardiac surgery, NYHA: New York Heart Association. Notes: CABG included on-pump CABG *n* = 80, off-pump CABG *n* = 73. Heart valve surgery included MICS *n* = 120.

**Table 3:** Multiple liner regression analysis of determining factors for achieving of independent walking.

	CABG Group (*n* = 153)		Heart Valve Surgery Group (*n* = 312)		Aortic Surgery Group (*n* = 102)	
	R^2^ = 0.499, *p* < 0.001		R^2^ = 0.698, *p* < 0.001		R^2^ = 0.606, *p* < 0.001	
	β [95%CI]	*p* Value	β [95%CI]	*p* Value	β [95%CI]	*p* Value
Age, year	0.057 [0.002–0.111]	0.041	−0.005 [−0.039–0.029]	0.776		
BMI, kg/m^2^	−0.064 [−0.221–0.093]	0.421				
NYHA functional classification, class			0.803 [0.355–1.251]	<0.001		
E/e’, cm/s	0.119 [−0.015–0.252]	0.082				
Comorbidity (presence)						
Renal disease	0.898 [0.177–1.619]	0.015	0.688 [0.139–1.238]	0.014		
Respiratory disease	0.327 [−0.367–1.021]	0.354	0.547 [0.013–1.080]	0.045		
Operative time, min			−0.001 [−0.007–0.006]	0.913	0.016 [−0.001–0.032]	0.061
Amount of bleeding, mL	0.001 [−0.001–0.001]	0.234			0.022 [−0.037–0.081]	0.458
Aortic cross clumping time, min					−0.008 [−0.050–0.034]	0.712
Cardiopulmonary bypass time, min					−0.006 [−0.034–0.023]	0.681
Emergency surgery (presence)	0.942 [−0.160–2.043]	0.093				
Length of intensive care unit stay, day	0.863 [0.499–1.226]	<0.001	1.326 [1.003–1.649]	<0.001	1.503 [0.947–2.058]	<0.001
Duration of mechanical ventilatory support, hour	0.010 [−0.014–0.033]	0.426	0.054 [0.031–0.078]	<0.001	−0.010 [−0.122–0.101]	0.858
Post-operative complications (presence)						
Cardiovascular complications	0.663 [−0.385–1.711]	0.213	0.835 [−0.192–1.479]	0.011	1.682 [−0.226–3.589]	0.083
Acute kidney injury			−0.453 [−1.761–0.855]	0.496	3.062 [0.451–5.673]	0.022
Delirium	0.155 [−0.903–1.214]	0.772	0.588 [−0.317–1.494]	0.202	1.052 [−0.770–2.874]	0.254
Respiratory complications	0.911 [0.269–1.555]	0.006	0.841 [0.303–1.378]	0.002	0.187 [−1.060–1.434]	0.767
Types of surgery (presence)						

Abbreviation: BMI: Body Mass Index, CABG: Coronary Artery Bypass Graft, E/e’: The ratio of mitral peak velocity of early filling to early diastolic mitral annular velocity, MICS: minimally invasive cardiac surgery, NYHA: New York Heart Association. Notes: CABG included on-pump CABG n = 80, off pump CABG n = 73. Heart valve surgery included MICS n = 120.

## Data Availability

The data that support the findings of this study are available from the corresponding author upon reasonable request.
